# Controlled Spalling of 4H Silicon Carbide with Investigated Spin Coherence for Quantum Engineering Integration

**DOI:** 10.1021/acsnano.4c10978

**Published:** 2024-10-29

**Authors:** Connor P. Horn, Christina Wicker, Antoni Wellisz, Cyrus Zeledon, Pavani Vamsi Krishna Nittala, F. Joseph Heremans, David D. Awschalom, Supratik Guha

**Affiliations:** † Pritzker School of Molecular Engineering, University of Chicago, Chicago, Illinois 60637, United States; ‡ Material Science Division and Center for Molecular Engineering, Argonne National Laboratory, Lemont, Illinois 60439, United States

**Keywords:** 4H-SiC, layer transfer, solid-state qubits, spin coherence, heterogeneous integration

## Abstract

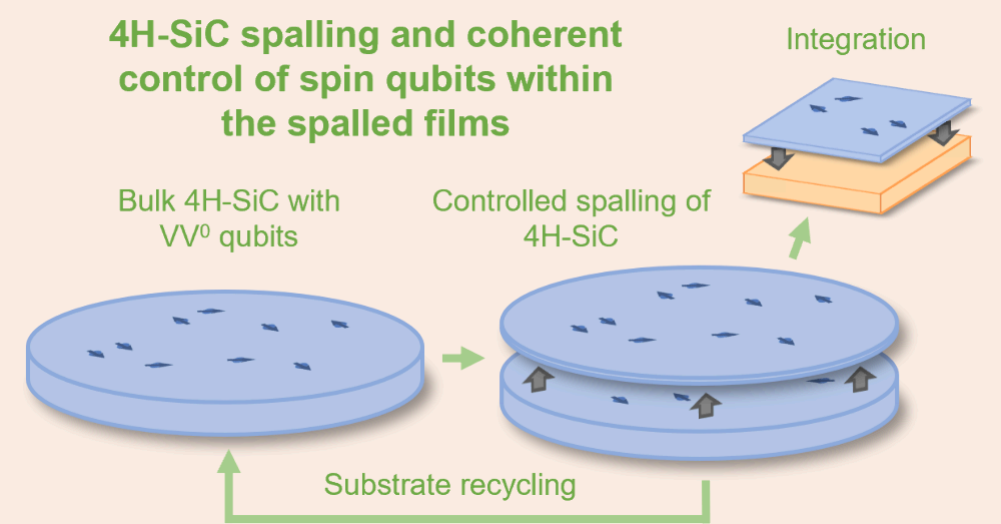

We detail scientific and engineering advances which enable the controlled spalling and layer transfer of single crystal 4H silicon carbide (4H-SiC) from bulk substrates. 4H-SiC’s properties, including high thermal conductivity and a wide bandgap, make it an ideal semiconductor for power electronics. Moreover, 4H-SiC is an excellent host of solid-state atomic defect qubits for quantum computing and quantum networking. Because 4H-SiC substrates are expensive (due to long growth times and limited yield), techniques for removal and transfer of bulk-quality films are desirable for substrate reuse and integration of the separated films. In this work, we utilize updated approaches for stressor layer thickness control and spalling crack initiation to demonstrate controlled spalling of 4H-SiC, the highest fracture toughness crystal spalled to date. We achieve coherent spin control of neutral divacancy (VV^0^) qubit ensembles and measure a quasi-bulk spin T_2_ of 79.7 μs in the spalled films.

Controlled spalling is a technique developed for extracting continuous films from a semiconductor substrate by triggered and deliberate propagation of a subsurface crack across the entirety of the chip or wafer.^1,2^ Stress is built up in the wafer subsurface by the deposition of an appropriate metal (stressor) layer on the wafer surface. The crack originates at the wafer edge and then propagates laterally at a depth of 10–50 μm to relieve this stress without a need for postconditioning (e.g., heat treatments). Spall depth can be modulated by engineering the stress field via the metal film deposition. A significant benefit of spalling is that the bulk-like properties of the exfoliated film are preserved^3−5^ since the crack depth is determined by an elastic stress field, rather than an intervention by ion implantation or by the deposition of heterogeneous layers at the separation interface. The principal breakthrough in spalling was made by Bedell et al., who introduced a controllable method for spalling using nickel films deposited under high tensile stress via sputtering or electroplating.^1^ This method has proven to be highly versatile, and to date has been used to spall Si, Ge, and III–V semiconductor wafers.^1,4−7^ Silicon wafers of up to 300 mm in diameter have been spalled.^1^ However, the materials spalled so far have been semiconductors with moderate to low fracture toughness and there have been no reports of successful spalling of more refractory, hard materials with a significantly higher fracture toughness.

One such material with a significantly higher fracture toughness is the technologically important semiconductor, silicon carbide (SiC), particularly the 4H polytype. 4H-SiC high-power electronics are being increasingly adopted in electric vehicles (4H-SiC MOSFET based inverters) and photovoltaic power management (4H-SiC high power diodes).^8,9^ 4H-SiC is also a leading wafer scale candidate for solid-state quantum coherent devices in quantum communications and sensing.^10,11^ Successful spalling of 4H-SiC creates two principal, distinct opportunities. First, a hindrance to further widespread adoption of 4H-SiC is the high cost of manufacturing substrates. High intrinsic defect density, challenging polytype control, high temperatures, and long growth times contribute to low yields and high substrate cost.^12,13^ Spalling offers a pathway for reusing a substrate multiple times if the spalled device layer can be integrated onto other substrates. Second, such layer removal via spalling motivates the heterogeneous integration of the resulting 4H-SiC membrane layers with other materials. This is particularly attractive for quantum technologies, where 4H-SiC has well characterized native defect-based qubits with long coherence times,^14^ and these native defects may be located and spalled to be integrated with silicon-based control electronics or embedded on photonic waveguides for applications in quantum communication.^15^

By demonstrating successful spalling of 4H-SiC, we have overcome the challenge of spalling an ultrahard material which requires 2.5 times greater strain energy than needed to spall GaN, the previous hardest material to be spalled.^6^ This result is enabled by scientific approaches taken in stressor layer design and spalling crack initiation (described in the Results and Discussion section). We present a controlled spalling-based solution for layer removal and transfer of few tens-of-microns thick films of single crystal 4H-SiC from bulk substrates. Bulk substrates are then repolished and can be reused to spawn further films for removal and transfer. We further show coherent spin control of a VV^0^ qubit ensemble in 4H-SiC with T_2_* and T_2_ of the same order of magnitude as in bulk substrates.

## Results and Discussion

Prior to spalling, a film of metal (typically Ni) is deposited onto the wafer to be spalled such that stresses in the metal layer give rise to an elastic stress field in the wafer subsurface region.^16^ The higher the fracture toughness of the wafer, the higher the thickness and stress of the metal film required to induce steady state spalling. The theoretical model which has proven to be valuable for predicting this thickness and stress is described by Suo and Hutchinson^17^ and enables calculation of the stress intensity factors K_I_ and K_II_ of a propagating crack within the substrate. Details and an application of this model have been described by Bedell et al.^4^ In summary, the crack originates at a free surface (usually the top surface of the semiconductor wafer) and propagates as a mixed mode crack (nonzero values of K_I_ and K_II_) plunging into the semiconductor. At a specific depth (predicted by the Suo and Hutchinson model) when K_II_ ∼ 0, the crack propagates in a direction that is on average parallel to the surface and spalls off a film of the semiconductor substrate attached to the metal stressor layer.

**Figure 1:**
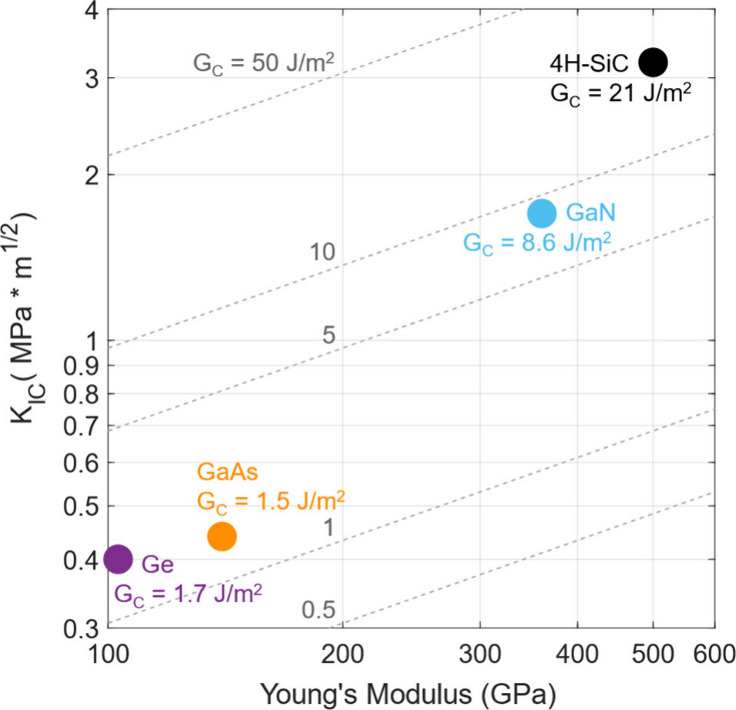
**Mechanical properties and strain energy comparison of spalled substrates.** The Ashby map depicts the mechanical properties of previously spalled substrate materials alongside 4H-SiC. The dashed lines denote equipotential G_C_ energies, plotted using eq 1. Note that the Poisson’s ratio used for the equipotential lines was 0.25, an average value among these semiconductors.

Successful spalling, however, requires one additional condition which is demarcated by the critical strain energy release rate G_C_ as described by Irwin and Orowan.^18,19^ The spalling crack propagation must release more strain energy than the energy which is required to break the bonds in the crystal. G_C_ is related to the material’s intrinsic fracture toughness (K_IC_) and is defined as
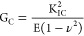
1Here, E is the Young’s modulus and ν is the Poisson’s ratio of the crack propagation medium. The semilog Ashby plot in Figure 1 illustrates the strain energy required for spalling 4H-SiC as compared to various materials which have previously been spalled. GaN was previously the most challenging material that had been spalled, whereas the 4H-SiC spalling demonstrated in this work requires almost 2.5 times more strain energy. The material properties used in this plot were gathered from a breadth of reported data^20−25^ as well as the previous papers^4−6^ on the spalling of each of the materials listed. Figure S1 gives further details on the required Ni metal stressor layers needed for spalling the selected materials. This previously unexplored level of strain energy per unit area applied to the metal–semiconductor spalling system poses new challenges critical to spalling of hard materials, notably (i) the thickness distribution of the metal film and (ii) the spalling crack nucleation. The scientific approaches used to address and overcome these challenges are described in this work and are expected to be applicable to the spalling of many other high fracture toughness semiconductors beyond 4H-SiC. We address below some of the key materials issues relevant to the spalling of such ultrahard materials.

### (i) Thickness Distribution of the Metal Film

The thickness distribution of the metal stressor layer holds considerable influence over the outcome of the controlled spalling process. Our studies have shown that the spalling fidelity of 4H-SiC is unpredictable if the thickness of the stressor layer at the substrate edges versus the substrate center varies by more than 10% from the intended distribution. This is because the fracture condition required for crack initiation and crack propagation are generally independent.^26^ Electroplated nickel has been used extensively^5,6,27,28^ as the metal of choice for spalling due to the availability of well characterized plating techniques, high deposition rate, and the ability to precisely control Ni stress through plating conditions.^29^ The Ni is electroplated onto a thin Cr/Au or Ti/Au seed layer that is initially sputtered on the wafer (see methods section for details). Although thickness uniformity can be excellent for sputtered Ni as compared to electroplating, the deposition rate (∼2 μm per hour) and maximum tensile stress (700 MPa)^4^ of sputtered Ni are undesirable and unacceptable, respectively, to attain the >20 μm thickness and ∼700–850 MPa stress needed to spall 4H-SiC (see Figure S1). These stress levels have been demonstrated via electroplating of Ni,^6^ but additional considerations are needed for ensuring desirable thickness distribution of the plated layer. Therefore, considerable effort was spent addressing a known aspect of electroplating called “current crowding” in which the deposit on the wafer edges can be up to 2–3 times thicker than the deposit thickness at the center.^30,31^

**Figure 2:**
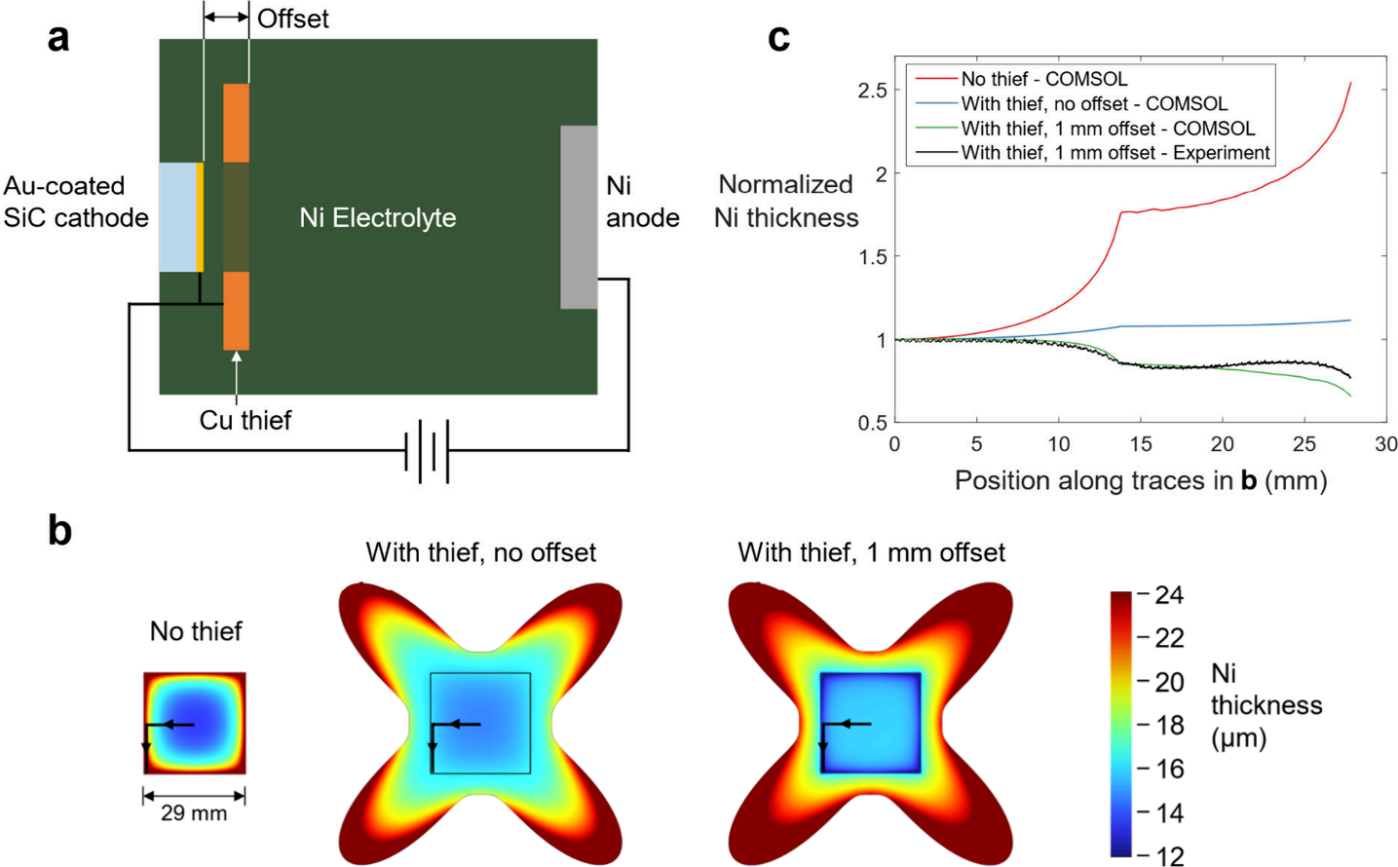
**Electroplating geometry and Ni thickness distribution. a** Top-down schematic of the electroplating geometry. The opening in the thief exactly matches the square shape of the SiC cathode. **b** COMSOL simulated Ni electroplating thickness distribution on 29 mm × 29 mm square substrates with and without thieves. Note that outer areas of the thieves exceed the plotted thickness range which is capped at 24 μm for better height resolution on the SiC surface. Current density averages 9 mA cm^–2^ on the SiC surface in all three cases. **c** Simulated and experimental measurements of Ni thickness along the traces in **b**. Values are normalized so that the thicknesses at the center of the substrates are coincident on the plot.

To address the problematic thickness nonuniformity of electroplated Ni, an auxiliary cathode which is known in the electroplating industry as a “thief”^32^ was designed and integrated into the electroplating setup. The thief adds additional surface area to the cathode and can be used to control current density at the edges of the target substrate by altering the current distribution. Figure 2a shows a diagram of the electroplating bath with the thief shorted to and coaxially offset from the Au-coated 4H-SiC cathode (which, as note earlier, acts as the seed for electroplating Ni). An iterative design process for the thief utilized finite element modeling in COMSOL to simulate the Ni electroplating thickness profiles on a 29 × 29 mm square substrate (typical size of 4H-SiC die used in this work) as a function of the size, shape, and position of a conductive thief surrounding it. Details of the electroplating parameters used in these simulations are given in the methods section.

Iteratively tuning the thief position (offset) and the parametric curve that describes the shape of the thief allows us to independently adjust: (1) the Ni thickness at the absolute center versus the edges of the target 4H-SiC substrate (such that the edges can be at least 30% thicker or thinner than the center), and (2) the Ni thickness around the edges of the square substrate by a similar factor at any segment along the perimeter. This enables optimization of the thickness at the corners versus the sides. Figure 2b, c shows examples of the thickness variation control possible with a thief having a square opening in the center and outer boundary defined by the equation
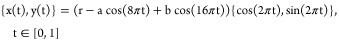
2where r, a, and b are positive constants that vary depending on the size of the square substrate. When paired with the chosen 1 mm offset, the Ni thickness hierarchy is as follows: center of chip > edge centers > corners. This scheme was chosen subsequent to the observation that square substrates nearly always start spalling from the corners, so if the corners are the last regions to reach the critical Ni thickness required for spalling, the rest of the substrate will already have sufficient stress from the Ni to easily propagate the spalling crack.

### (ii) Spalling Crack Nucleation

**Figure 3:**
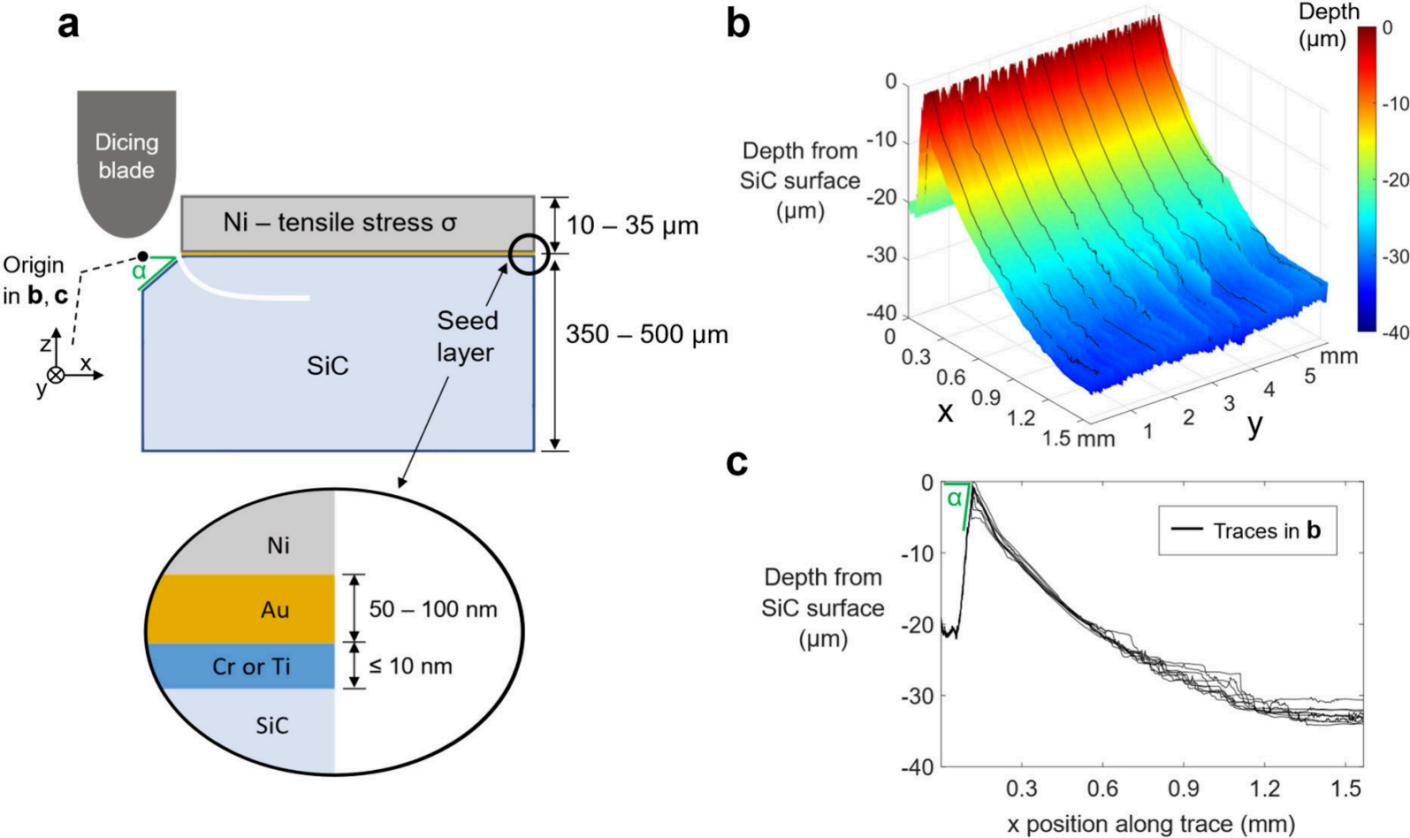
**Spalling crack initiation into 4H-SiC. a** Diagram showing the spalling geometry for crack initiation and propagation. **b** 3D laser scanning confocal microscope map taken at the crack initiation edge of a 4H-SiC substrate after spalling. Trench for crack initiation is present from x = 0 to 0.15 mm, beyond which the spalling crack is mapped. **c** Black line traces on the 3D map in **b** are merged and plotted as spall depth versus x position. Trench angle α is measured to be 29°.

Another concern for the spalling of ultrahard materials is that crack initiation–the prerequisite to steady state spalling–was found to be unreliable when utilizing controlled spalling techniques which have been established previously. The conventional method by which spalling crack initiation is made into Si and other semiconductors of similar toughness is to make the Ni abruptly discontinuous away from the substrate edge.^1,4^ The stress concentration where the edge of the Ni meets the substrate is then high enough such that a crack can initiate either spontaneously when a certain Ni thickness is reached, or with external force from a handle layer of tape which is applied on top of the Ni and pulled upward. In this case, no modification of the substrate is needed to initiate a spalling crack, i.e. α = 0° as defined by the angle of the substrate from horizontal at the edge of the Ni, as shown in Figure 3. For 4H-SiC however, we found that crack initiation fails when using this technique. Rather than inducing a spalling crack, failure ultimately occurs by delamination at the Au/Ni interface (see Figure S2) when the electroplated Ni far exceeds (>4x) the Suo-Hutchinson critical thickness for spalling.^17^ The adhesion between the 4H-SiC substrate and the Cr or Ti was consistently observed to be strong and rarely a source of delamination.

A solution to this crack initiation problem was found by modifying the 4H-SiC substrate such that α > 0° as defined in Figure 3a. In practice, we chose to cut a shallow trench at the edge of the 4H-SiC substrate with a standard dicing saw after seed layer deposition, following which the Ni electroplating was carried out. By adjusting the cut depth, we used the elliptically shaped blade tip to deliberately vary α angles from 8° - 90° and initiate spalling at the top surface of the 4H-SiC substrate directly adjacent to the trench. Figure 3b, c depicts 3D and 2D laser confocal microscope scanned surface profiles at the crack initiation edge of a spalled 4H-SiC substrate with α ∼ 29°. The trench spans from x = 0 to 0.15 mm, and then for x > 0.15 mm the spalling crack initiates from the 4H-SiC surface, plunging downward into the substrate until the equilibrium spall depth is reached. This method of inducing a spalling crack with an angled trench appears to be counter to the expectations of other relevant studies on crack initiation at the edge of stepped boundaries.^26,33^ These suggest that as compared to larger α angles, α = 0° should concentrate the K_I_ stress intensity most highly where the edge of the Ni meets the substrate and most strongly favor crack initiation. However, we consistently observe that such an incision leads to successful spalling of 4H-SiC. The reason for this is not clearly understood. One speculation may be the formation of incipient cracks or damage by the grit of dicing saw that reduces the threshold for crack initiation, though we note that manual cleavage of the edge to create α = 90° also enables successful 4H-SiC spalling, suggesting that the advantage of the dicing cut is not unique. Once the crack has been initiated, it is then free to propagate through the 4H-SiC substrate according to Suo and Hutchison model.^17^

### Physical Characterization of Spalled Substrates and Films

**Figure 4:**
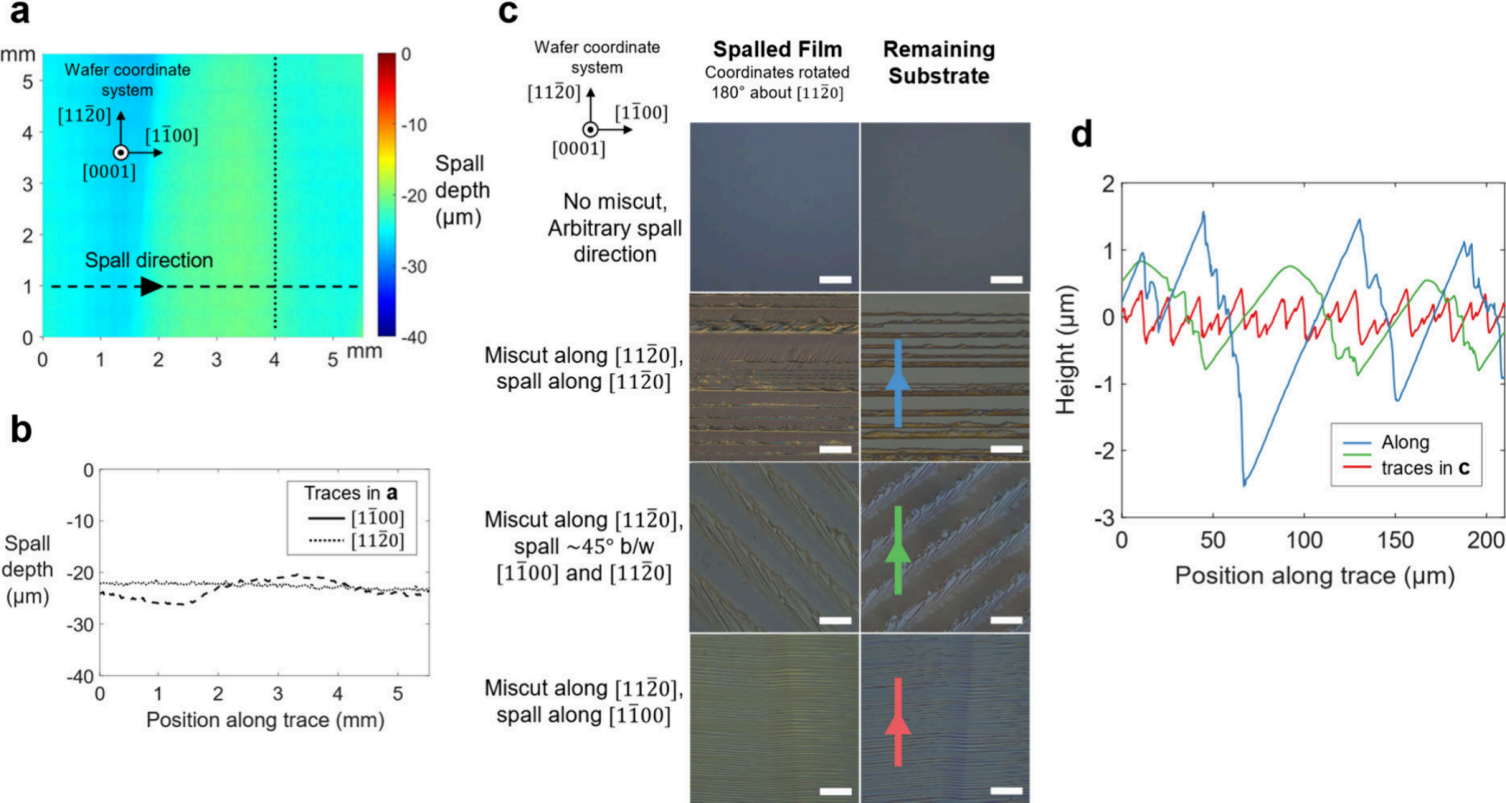
**Spalled 4H-SiC film and substrate surface morphology. a** 3D laser scanning confocal microscope map of the surface of a remaining substrate after spalling. Absolute spall depth and tilt are calibrated by deliberately unspalled regions outside the boundaries of the map shown. Wafer coordinate system shown assumes a nonmiscut crystal. **b** Profiles of spall depth versus position for the line traces in **a**. **c** Optical differential interference contrast images of various spalled films and substrates. Blue, green, and red colored traces indicate the scan direction of the profiles in **d**. Wafer coordinate system shown assumes a nonmiscut crystal. Scale bars, 100 μm. **d** Laser scanning confocal microscope profiles of spall depth variation versus position for the line traces in **c**, centered about the mean surface heights.

The 4H-SiC substrates spalled in this work ranged from 5 × 5 mm to 29 × 29 mm squares cut from 100 mm or 150 mm wafers. The decision to spall square dies instead of full wafers reflects the high cost of 4H-SiC wafers and not any anticipated new challenges with spalling larger substrates. In Figure 4, 3D laser scanning confocal microscopy studies were carried out to further explore the surface morphology of the spalled face. A 5.5 × 5.5 mm area near the center of a spalled substrate is investigated in Figure 4a, b. The profiles reveal that spall depth along the direction of crack propagation ([11̅00]) is more variable (∼6 μm peak-to-peak undulation) than spall depth perpendicular to the direction of crack propagation ([112̅0]) (<1 μm fluctuation). This outcome can imply a slight variability in the speed or angle of crack propagation which in turn causes the crack depth to waver.^6^ For applications which require a more homogeneous spall thickness, a mechanical system can be adopted to better control the spalling crack speed and angle to improve this nonuniformity.^2^

4H-SiC substrates that were both on-axis (c-plane and m-plane) as well as off-axis (c-plane with 4-degree miscut toward [112̅0]) were used in this study, since the latter orientation is often used in power electronics applications. On-axis c-plane (0001) and m-plane (101̅0) 4H-SiC were both found to yield smooth corrugation-free surfaces as pictured in Figure 4c, establishing that both of these planes exhibit favorable cleavage for spalling. When spalling the miscut substrates, matching corrugations were observed on the surfaces of the remaining substrates and spalled films, with amplitude and spall angle dependent on spall direction as plotted in Figure 4d. The perpendicular-to-miscut spall ([11̅00] spall direction) had the lowest roughness: the mean ± standard error of the maximum peak-to-peak height measured across ten line profiles was 0.927 ± 0.015 μm. It is well-known in spalling literature that semiconductors with ionic character (GaAs, InP) tend to spall along specific crystal planes, whereas elemental semiconductors are not as bound to this tendency.^1,2,5,7^ SiC has comparable ionicity to GaAs (electronegativity difference 0.7 for SiC and 0.5 for GaAs),^34^ which explains the sawtooth-like spalling of the miscut substrates.

### Substrate Reuse and Integration of Spalled Films on Carrier Substrates

To demonstrate substrate reuse, a selection of previously spalled substrates was repolished using a standard lapping and chemical mechanical polishing procedure for 4H-SiC (see methods). The substrates were then respalled to establish that there are no unforeseen problems caused during surface reconditioning which would otherwise prevent further spalling. In total, the thickness reduction of the initial 4H-SiC substrate due to spalling and repolishing can be limited to the maximum spall depth + approximately 15 to 20 μm due to the lapping and polishing process. Images of the repolished and respalled substrates are included in Figure S3.

**Figure 5:**
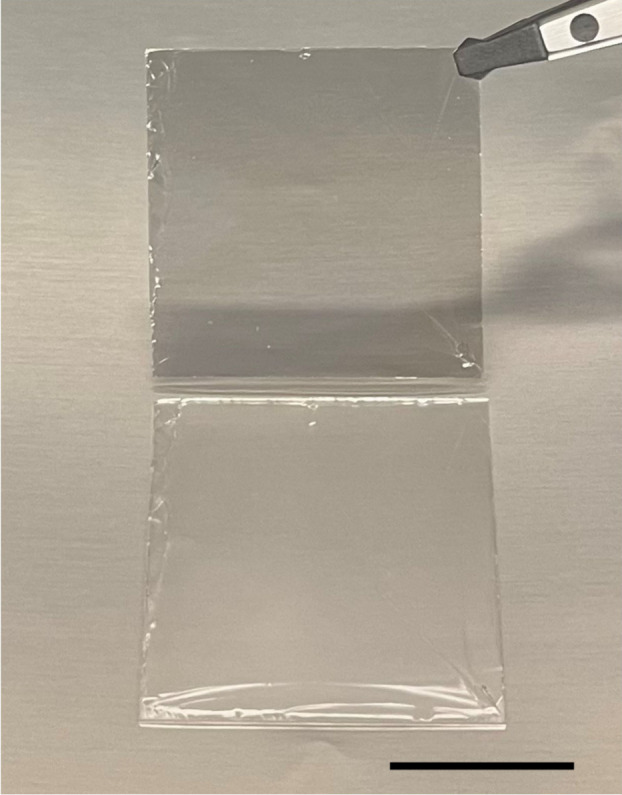
**Picture of spalled 4H-SiC and remaining substrate.** The ∼30 μm thick spalled film is held with a tweezer above the corresponding substrate which it originated from.

Once the metals for spalling are etched away, the freestanding spalled 4H-SiC (typically 10–50 μm thick) is still rigid enough to be handled easily (see Figure 5) for transfer and bonding to a handle substrate. For example, we routinely bond the spalled films to a silicon wafer using a 25 μm thick epoxy-based die attached film from AI Technology Inc., as pictured in Figure S3. This bonding does not require any intermediate manipulations or carriers to be realized; the Ni is simply wet etched away from the spalled film and the film then pressed onto the bonding tape and heated to 120 °C to cure the epoxy bond.

### Measurement of Qubit Properties in Spalled Films

As noted in the introduction, spalled 4H-SiC is an attractive approach for heterogeneously integrating 4H-SiC spin-qubit based quantum coherent devices with silicon. Optically active defect spin qubits in 4H-SiC, including transition metal ions such as vanadium,^35^ and vacancy complexes such as the nitrogen-vacancy center (NV)^36^ and the divacancy (VV)^37^ are widely investigated for quantum computing,^10^ networking,^38^ and sensing.^39^ The spalling process seeks to overcome scalability challenges of these technologies by creating transferable thin films from semiconductor qubit hosts. We characterize the coherence properties of spin qubits subjected to spalling to infer the quality of native spalled films for quantum applications.

**Figure 6:**
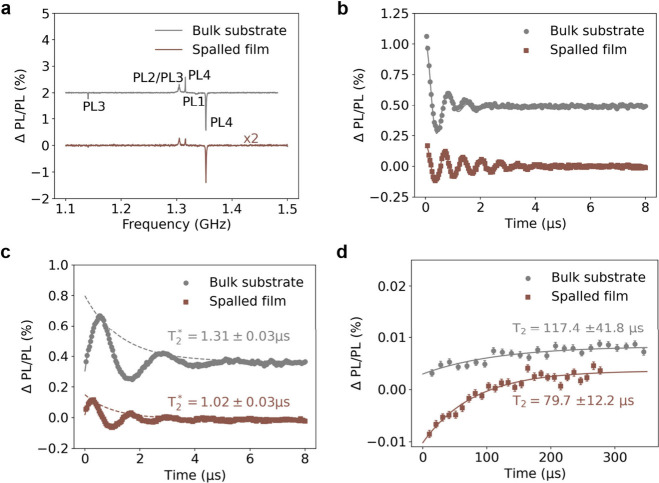
**Comparison of 4H-SiC VV**^**0**^**qubit properties between bulk wafer and spalled film. a** Continuous-wave (CW) ODMR spectra for the HPSI bulk wafer and spalled film measured as a function of microwave frequency and detected as a normalized change in photoluminescence intensity. **b** Rabi oscillations of the basal (PL4) divacancy in HPSI bulk wafer and spalled film. The driving frequency is resonant to the PL4 dip at 1.353 GHz in the CW ODMR or pulsed ODMR spectra. **c** Ramsey decay of PL4 divacancy defects showing a film T_2_* ∼ 78% of the bulk. The dashed lines illustrate the Ramsey decay envelope. **d** Hahn echo decay measurements showing a film T_2_ ∼ 68% of the bulk. In b,c,d, solid lines are fits, and error bars are standard errors corresponding to 95% confidence intervals. All measurements are performed at a temperature of 8 K.

To benchmark the performance of spalled 4H-SiC for quantum applications, we study the spin properties of neutral-divacancy defects (VV^0^) in spalled high purity semi-insulating (HPSI) 4H-SiC. Figure 6a shows a continuous-wave optically detected magnetic resonance (ODMR) spectra in the absence of an applied external field. Both samples show a pronounced resonance of PL4 divacancies centered at 1.353 GHz. Photoluminescence data is additionally included in Figure S4. Coherent spin control of the PL4 ensemble in the spalled film and bulk substrate was then performed to compare spin coherence times. In Figure 6b, Rabi oscillations are observed in the film by sweeping the duration of a single microwave pulse. Additionally, a Ramsey pulse sequence is used to characterize the spin T_2_* as shown in Figure 6c. A T_2_* = 1.31 ± 0.03 μs was measured in the bulk wafer, consistent with the literature values for VV^0^ ensembles at this temperature,^37^ and a T_2_* = 1.02 ± 0.03 μs was measured in the film. Similarly, we perform Hahn echo measurements in Figure 6d to characterize the coherence time T_2_ between the two samples and find a slightly shorter T_2_ = 79.7 ± 12.2 μs in the film compared to T_2_ = 117.4 ± 41.8 μs in the bulk sample. The lower film T_2_* and T_2_ are attributed to additional dephasing caused by broadening of the ensemble spin resonances. However, these values are sufficiently high for us to regard the film as quasi-bulk, and demonstrate the viability of these spalled films as a defect center qubit host platform. The primary mechanism for the resonance broadening is currently unclear. It is known that broadening can be caused by the presence of extended defects such as dislocations^40^ or elastic strain^41^ in the crystal. It is possible that such degradations occur after the bending and manipulation of the 4H-SiC film during spalling. However, a full theoretical treatment correlating the magnitude of resonance broadening to these effects is beyond the scope of this work. A future detailed study which correlates coherence properties to microstructural strain in spalled 4H-SiC could provide guidance on how to optimize the quality of spalled films.

## Conclusion

Controlled spalling of 4H-SiC has been successfully demonstrated on a wide variety of substrate types and sizes. Reliable spalling of this high fracture toughness material is made possible by finely tuning the stressor layer thickness distribution and spalling crack initiation into the 4H-SiC. Thickness nonuniformity across an entire spalled film is currently ∼6 μm peak-to-peak, limited primarily by further advancements needed in regulation of the peeling process. Intrinsic roughness of 4-degree miscut substrates can be kept under 1 μm peak-to-peak by spalling perpendicular to the miscut direction. Heterogenous integration and substrate reuse show promise, with more complicated schemes to be pursued in future work. An initial demonstration of coherent spin control of a VV^0^ ensemble in spalled 4H-SiC yields a spin T_2_* which is similar (∼78%) to the bulk value and a spin T_2_ which is likewise similar (∼68%) to the bulk value, sufficient to motivate future exploration of spalled films incorporated with on-chip silicon photonics. Other goals for future work include spalling full 150 mm and 200 mm wafers of 4H-SiC as well as exploring the spalling of 4H-SiC substrates with prefabricated power devices on the wafer surface.

## Methods

### 4H-SiC Substrate Preparation

The 4H-SiC substrates used in this work were grown via physical vapor transport by ST Microelectronics N.V., GlobiTech, Inc., and Wolfspeed, Inc. Wafers spanned from 350 to 500 μm thick and were comprised of n-type (0001) with 4-degree off-axis miscut toward [112̅0], n-type (0001) on-axis, and high purity semi-insulating (HPSI) (0001) and (101̅0) on-axis substrates. Immediately following a cycle of SC-1, SC-2, and 10:1 buffered oxide etchant (BOE) cleaning steps, a seed layer of ≤10 nm of Cr or Ti and then 50–100 nm of Au was deposited in an AJA Orion UHV Sputtering System with 2-in. targets. The Cr or Ti was deposited at 100 W RF in 5 mTorr Ar, while the Au was deposited at 100 W DC in 5 mTorr Ar. All wafers were then diced into square dies, and the trench for crack initiation was also cut at this time. These processes utilized a model 7122 Advanced Dicing Technology (ADT) dicing saw with a 150 or 200 μm thick resin blade containing 46 μm diamond grit.

### Ni Electroplating Conditions

The general procedure for Ni stressor layer deposition and subsequent spalling has been described thoroughly elsewhere.^1,4^ Ni electroplating baths used in this work contained 300 g L^–1^ NiCl_2_ · 6 H_2_O, 30 g L^–1^ H_3_BO_3_, and 10–20 g L^–1^ NH_4_Cl with current densities ranging from 8–30 mA cm^–2^. All depositions took place at room temperature. The stress of the electroplated Ni was modulated via the NH_4_Cl concentration and current density used. Higher NH_4_Cl concentrations and higher current densities resulted in higher stress deposits. Ni stress was measured using the bent strip method as defined under ASTM Standard B975 with products from Specialty Testing and Development Company. Electroplating baths ranged in size from 120 mL to 2 L, depending on the size of sample to be spalled and the quantity of fluid needed to keep the bath temperature from rising by more than 2 °C at high current densities. The 120 mL baths were used for the 5 × 5 mm square substrates and simply contained a 7/8 in. spin bar at 150 rpm to agitate the bath. The 2 L baths were used for the 29 mm × 29 mm square substrates and employed a Watson-Marlow model 323E peristaltic pump to circulate the solution at ∼1 L min^–1^. Spalling handle layers included polyimide tape with silicone adhesive or Revalpha Heat Release Tape by Nitto Denko Corporation.

### Ni Electroplating COMSOL Simulations

A replica of the experimental electroplating geometry was created in COMSOL, and the Secondary Current Distribution physics interface of the Electrochemistry Module was used to simulate the Ni electroplating dynamics. At all electrode surfaces, the Ni reaction was defined with an equilibrium potential of −0.26 V and a Butler–Volmer kinetics expression was defined for the Ni reaction. The exchange current density for Ni was set to 0.1 A m^–2^, while the anodic and cathodic transfer coefficients were both set to 0.5. At the 4H-SiC + thief cathodes, an additional hydrogen evolution reaction was defined to have 0 V equilibrium potential and a cathodic Tafel kinetics expression. The exchange current density for H was set to 2 × 10^–5^ A m^–2^, while the cathodic Tafel slope was set to −118 mV. Electrolyte conductivity was set to 10 S m^–1^. The study steps involved a current distribution initialization and then a time dependent step in which Ni was deposited for a set amount of time. Because neither a deforming geometry nor a tertiary current distribution was used, the deposition rate is constant and thus the chosen plating time is arbitrary.

### Optical Profiling and Imaging

For post processing and characterization, all Ni thickness and spall depth area scans and line profiles (Figure 2, 3 and 4) were measured with the 20x lens of a Keyence VK-X1000 Laser Scanning Confocal Microscope. Automated image stitching was used for large area scans. Optical microscope images were taken with a differential interference contrast (DIC) enabled Olympus BX60 reflected light microscope and Tucsen MIchrome 5 Pro digital camera.

### Lapping and Chemical Mechanical Polishing of 4H-SiC

Spalled 4H-SiC substrates were mounted to a granite puck using Crystalbond 509 wax. The puck was then flipped to face downward on a polishing pad wetted with slurry. Initial lapping utilized a 3 μm diamond grit slurry to remove the spalling divot and replanarize the substrates. Next, a 0.5 μm diamond grit slurry removed the surface damage from the 3 μm grit, removing an additional 10 μm of material. Finally, a colloidal silica slurry was used in the chemical mechanical polishing process to ultimately achieve an epi-ready surface, removing <2 μm of material.

### VV^0^ Qubit Measurements

The VV^0^ are excited with below bandgap light from a 905 nm (1.37 eV) diode laser in a cryostat. The bulk sample and spalled film are both measured at a nominal temperature of 8 K. For ODMR measurements, optical emission from the sample is filtered through a 1000 nm long pass filter and 1050 nm dichroic, focused on a fiber-coupled photodiode (Femto OE200-IN1). A lock-in amplifier (Signal Recovery 7265) is used in detection with the reference oscillator corresponding to square wave amplitude modulation of the microwave drive at 503 Hz and 50% duty cycle. For the Rabi (Figure 6b), Ramsey (Figure 6c), and Hahn echo (Figure 6d) measurements, an optical pulse is used to initialize the spin state, microwave pulses coherently manipulate the spins, and a second optical pulse causes a readout of spin-state dependent luminescence. In both cases the driving field on the sample is aligned to the *c*-axis. The Rabi and Ramsey data were fit to exponentially decaying sinusoids. The Hahn echo recovery was fit to an exponential function.

## Supporting Information Available

Critical spalling conditions, crack initiation failure, spalled film and substrate processing, and photoluminescence scans on the divacancy ensembles (PDF)
