# Supplementary material for: Controlled Spalling of 4H Silicon Carbide with Investigated Spin Coherence for Quantum Engineering Integration
Source: ACS Nano. 2024 Oct 29;18(45):31381–9. doi: 10.1021/acsnano.4c10978 (PMC11562792; doi:10.1021/acsnano.4c10978)
Supplement: Supplementary file 1 — nn4c10978_si_001.pdf [file nn4c10978_si_001.pdf]

Supplementary to Controlled Spalling of 4H Silicon Carbide with Investigated Spin Coherence for Quantum Engineering Integration

*Connor P Horn[†,‡], Christina Wicker[†,‡], Antoni Wellisz[†], Cyrus Zeledon[†], Pavani Vamsi Krishna Nittala[† #], F Joseph Heremans[†,‡], David D Awschalom[†,‡], Supratik Guha[†,‡]**

† Pritzker School of Molecular Engineering, University of Chicago, Chicago, IL 60637 USA

‡ Material Science Division and Center for Molecular Engineering, Argonne National Laboratory, Lemont, IL 60439 USA

* Please address correspondence and requests for materials to S. G. (email: guha@uchicago.edu)

Now at Micron Technology, Inc., Boise, ID 83716 USA

1. Minimum Ni stressor conditions for spalling different substrate materials.

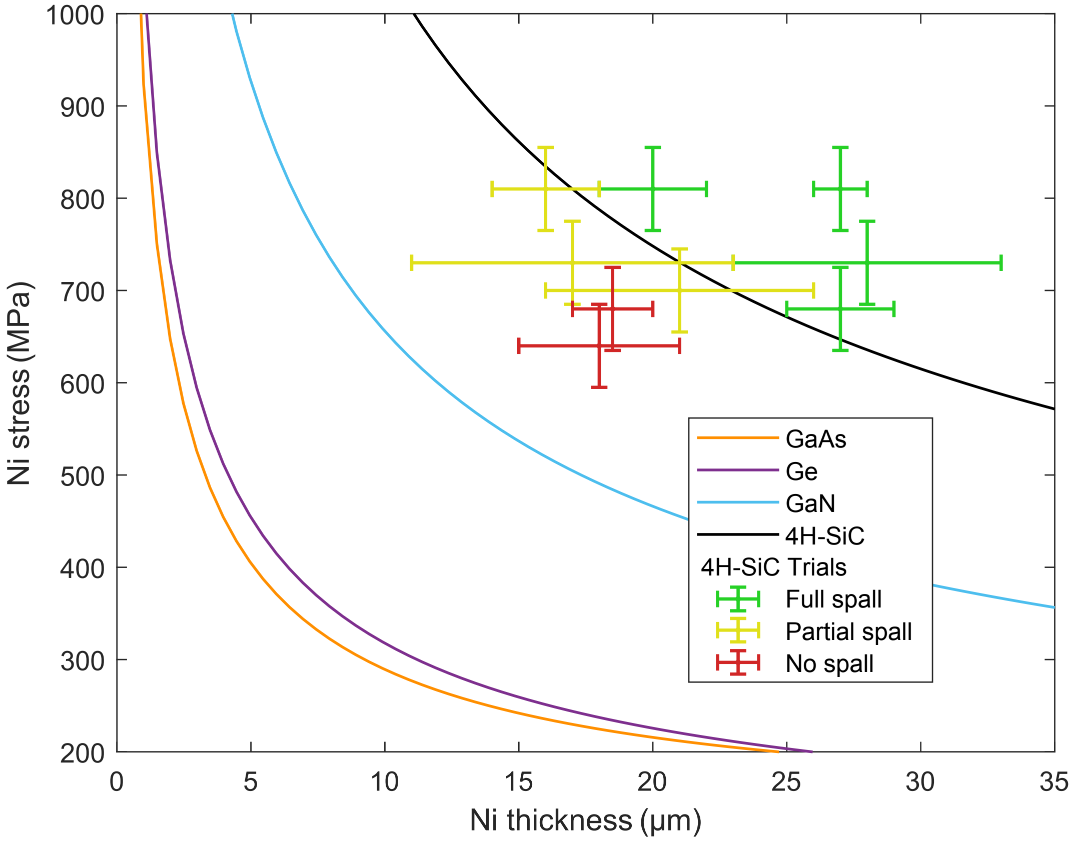

Figure S1. The curves for GaAs, Ge, and GaN are fit to the Suo and Hutchinson mathematical model[1] using data and extracted material properties found in previous spalling work[2–4] and independent experiments.[5–8] These curves represent the critical minimum Ni thickness and stress needed for controlled spalling. The 4H-SiC curve is also fit to the Suo and Hutchinson model; the Young's modulus (500 GPa) and Poisson's ratio (0.16) are chosen as average values from literature[8–10] and the fracture toughness (3.20 MPa $m^{1/2}$) is fit so that the curve agrees with the experimental trials from this work. Other relevant material properties used in fitting the curves include the Young's modulus of Ni (200 GPa) and the Poisson's ratio of Ni (0.31).[2] All substrate thicknesses were set to 1000 μm to reflect that the substrates are held down either with double sided tape or a vacuum chuck, therefore increasing this variable to near-infinite.[2] The experimentally determined 4H-SiC fracture toughness of 3.20 MPa $m^{1/2}$ in this work agrees well with a study on 4H-SiC single crystals

using indentation tests, which reports fracture toughnesses ranging from 3.15 MPa $m^{1/2}$ to 3.33 MPa $m^{1/2}$ for indentations on the prismatic $(10\bar{1}0)$ and basal (0001) planes, respectively.[9]

Data from the experimental trials in this work is plotted using the green, yellow, and red error bars. Ni thickness was set by plating time and stress was modulated as defined in the methods section. In many cases, numerous trials were run at a particular condition, but are not plotted to avoid crowding the plot with duplicate information. Ni thickness was measured with a 3D laser scanning confocal microscope to < 0.5 μm accuracy. The wider thickness ranges in the plot represent the nonuniformity in Ni electroplating thickness on the substrate, specific to the electroplating parameters for each trial. Since Ni stress / wafer bowing could not be accurately measured on the small square-shaped samples, proxy measurements were done in a secondary plating bath using the bent strip method (ASTM Standard B975). Various bath chemistries and current densities were characterized and a standard deviation of 23 MPa was calculated. Error bars for the Ni stress are plotted to include two standard deviations above and below the mean, to reflect a 95% confidence interval. The relatively large (~ 90 MPa) confidence interval also accounts for an observed day-to-day variability in Ni stress potentially due to several factors including changes in pH, NH_4Cl concentration, temperature, contamination, etc. The Suo and Hutchinson curve for 4H-SiC is fit so that it completely encapsulates the fully spalled cases and encapsulates approximately a quarter of the partially spalled Ni thickness range, while excluding all the no-spall cases.

2. Details of Ni delamination during electroplating.

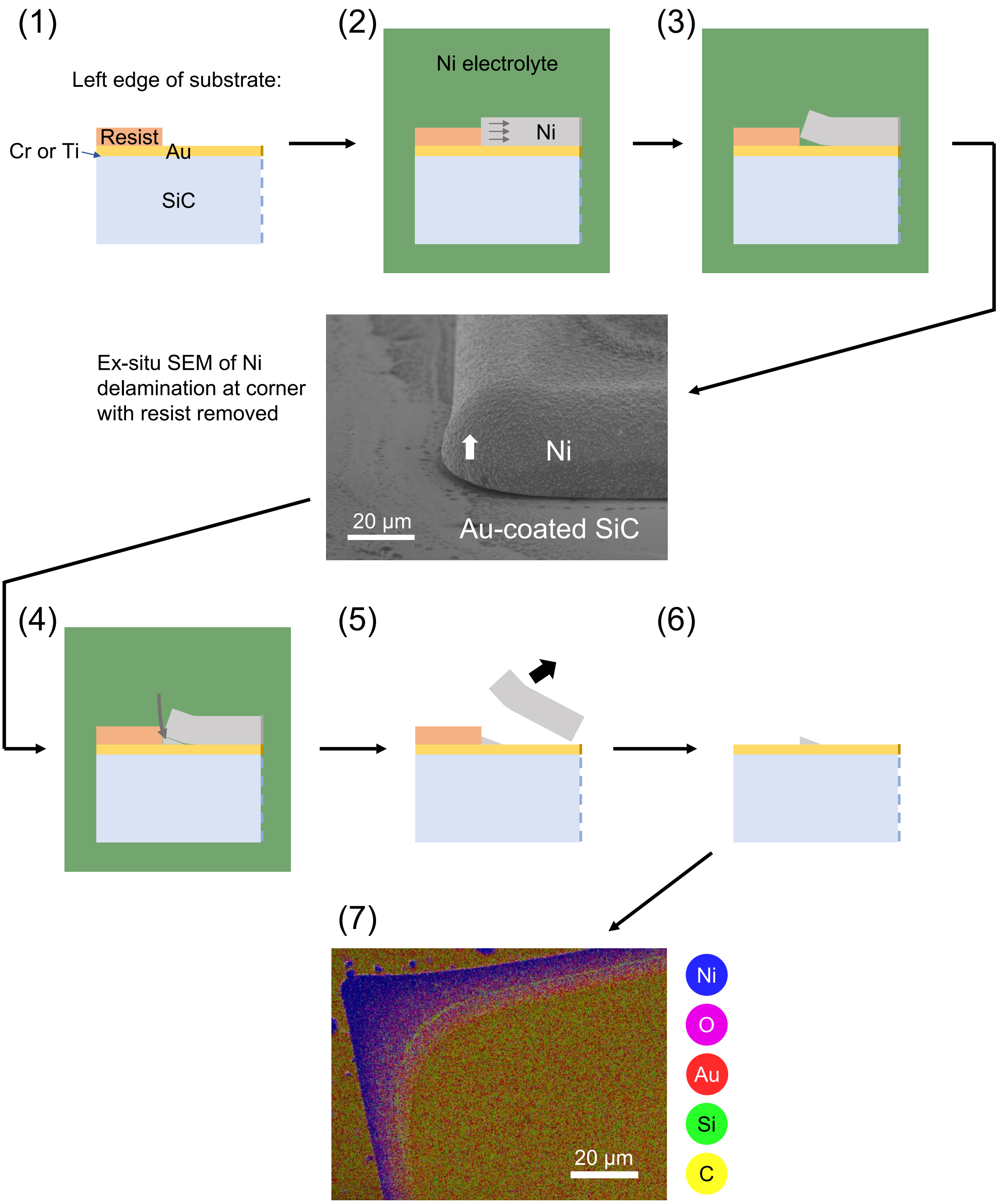

Figure S2. During electroplating of the Ni layer, which is under a high intrinsic tensile stress, we observe that the Ni will partially delaminate around the perimeter of the area it is covering. However, when that delamination happens between metal surfaces (in this case the Ni and the Au from the sputtered seed layer), Ni ions will re-deposit into the wedge which opens up

underneath the delaminating Ni. This re-deposition effect prolongs and can even eliminate the possibility of full delamination of the Ni from the Au-coated 4H-SiC substrate. The timeline is as follows:

(1) Polymer resist is applied on the perimeter of the Au-coated 4H-SiC substrate to electrically mask the Ni plating and define the edge of the Ni where the spalling crack should initiate.[2,11] (2) Ni is electroplated onto the substrate, and the high intrinsic tensile stress of the Ni (three arrows indicate direction) causes (3) the Ni to slightly delaminate from the Au at the edge. Where the Ni lifts off the Au, (4) a new deposit of Ni forms on the exposed Au surface. (5) If the plated Ni film is peeled off / fully delaminates, the Ni deposit on the newly exposed gold remains, appearing to have better adhesion to the Au than the underside of the Ni film. (6) The resist is washed away and (7) the elements on remaining surface are characterized via an energy dispersive X-ray spectrometer (EDS) in a JEOL IT800HL SEM and analyzed using Oxford Instruments AZtec software. The graduated Ni deposit around the perimeter of the corner is due to the original Ni film lifting up at the edges and new Ni plating on the Au underneath. The oxygen is from the native oxide on the Ni, and the Si and C from the 4H-SiC substrate are detectable through the Au layer. The Cr from the seed layer is too thin to be detected.

3. Heterogenous integration and substrate reuse of spalled 4H-SiC.

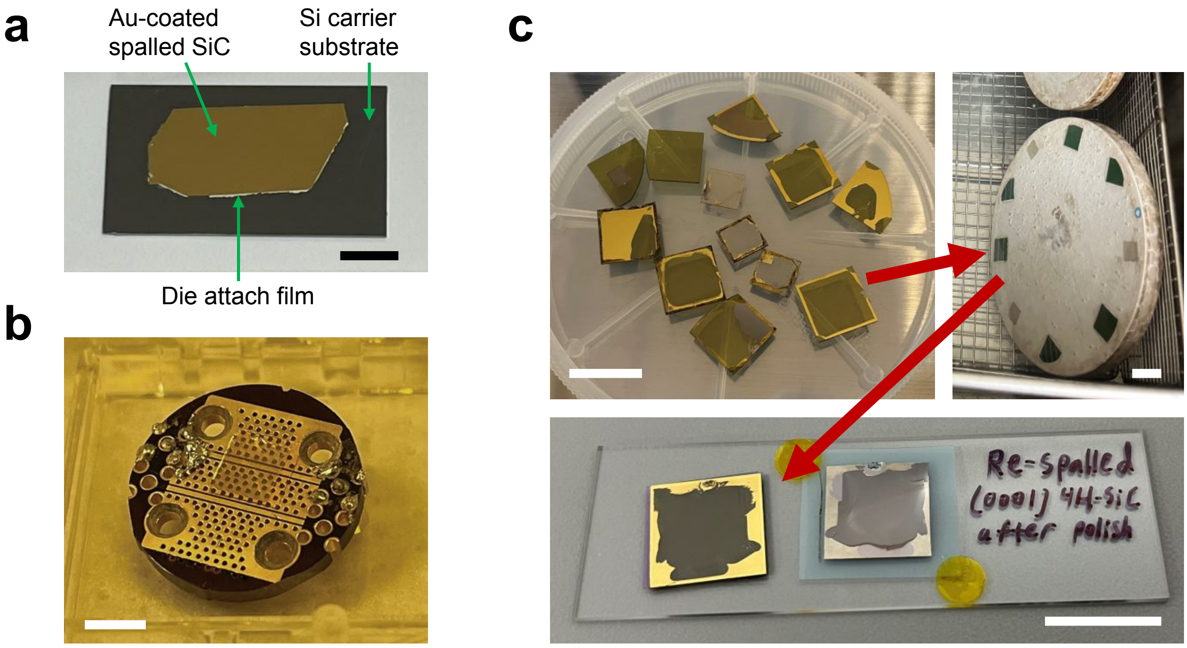

Figure S3. a Spalled 4H-SiC is bonded to a silicon carrier chip using an epoxy-based die attach film. The gold from the seed layer was not etched away so that the spalled film is highly visible. Scale bar, 5 mm. **b** HPSI spalled 4H-SiC film is mounted over a stripline on a printed computer board using double sided polyimide tape for the application of microwave pulses during qubit manipulation. Scale bar, 5 mm. **c** A selection of previously spalled substrates is mounted to a granite puck (top right) and then undergoes lapping and polishing to return the surfaces to their original pre-spalling polish. Substrates are then re-spalled with no complications. Note that these substrates were spalled before the crack initiation methods were improved, which explains the irregularly shaped spall regions pictured. Scale bars, 15 mm.

4. Photoluminescence spectrum of bulk wafer and spalled film.

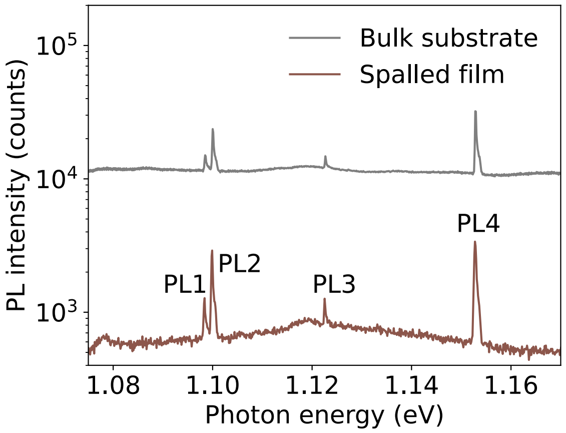

Figure S4. Sharp lines from 1.09 – 1.16 eV are present in both the bulk wafer and spalled film, corresponding to the zero phonon lines of PL1 – PL4 divacancies. This data is acquired from a different substrate than used in the main text. The spectra are recorded using a spectrometer with an InGaAs detector. The spectrometer grating is 600 g mm^{-1} and the slit width is 25 µm. No additional optical broadening is observed in the film as zero-phonon lines on both samples are narrower than the spectrometer resolution limit.

References

(1) Suo, Z.; Hutchinson, J. W. Steady-State Cracking in Brittle Substrates beneath Adherent Films. *Int. J. Solids Struct.* **1989**, *25* (11), 1337–1353.

(2) Bedell, S. W.; Fogel, K.; Lauro, P.; Shahrjerdi, D.; Ott, J. A.; Sadana, D. Layer Transfer by Controlled Spalling. *J. Phys. Appl. Phys.* **2013**, *46* (15), 152002.

(3) Sweet, C. A.; Schulte, K. L.; Simon, J. D.; Steiner, M. A.; Jain, N.; Young, D. L.; Ptak, A. J.; Packard, C. E. Controlled Exfoliation of (100) GaAs-Based Devices by Spalling Fracture. *Appl. Phys. Lett.* **2016**, *108* (1), 011906.

(4) Bedell, S. W.; Lauro, P.; Ott, J. A.; Fogel, K.; Sadana, D. K. Layer Transfer of Bulk Gallium Nitride by Controlled Spalling. *J. Appl. Phys.* **2017**, *122* (2), 025103.

(5) Cheng, Y.; Cai, D.; Wang, H.; Wu, J.; Liu, X.; Zhang, G.; Yu, T. Anisotropic Fracture Toughness of Bulk GaN. *Phys. Status Solidi B* **2018**, *255* (5), 1700515.

(6) Ericson, F.; Johansson, S.; Schweitz, J.-Å. Hardness and Fracture Toughness of Semiconducting Materials Studied by Indentation and Erosion Techniques. *Mater. Sci. Eng. A* **1988**, *105–106*, 131–141.

(7) King, S. W.; Antonelli, G. A. Simple Bond Energy Approach for Non-Destructive Measurements of the Fracture Toughness of Brittle Materials. *Thin Solid Films* **2007**, *515* (18), 7232–7241.

(8) Jain, A.; Ong, S. P.; Hautier, G.; Chen, W.; Richards, W. D.; Dacek, S.; Cholia, S.; Gunter, D.; Skinner, D.; Ceder, G.; Persson, K. A. Commentary: The Materials Project: A Materials Genome Approach to Accelerating Materials Innovation. *APL Mater.* **2013**, *1* (1), 011002.

(9) Eswar Prasad, K.; Ramesh, K. T. Hardness and Mechanical Anisotropy of Hexagonal SiC Single Crystal Polytypes. *J. Alloys Compd.* **2019**, *770*, 158–165.

(10) Chen, J.; Fahim, A.; Suhling, J. C.; Jaeger, R. C. A Study of the Elastic Constants of 4H Silicon Carbide (4H-SiC). In *2019 18th IEEE Intersociety Conference on Thermal and Thermomechanical Phenomena in Electronic Systems (ITherm)*; 2019; pp 835–840.

(11) Bedell, S. W.; Shahrjerdi, D.; Hekmatshoar, B.; Fogel, K.; Lauro, P. A.; Ott, J. A.; Sosa, N.; Sadana, D. Kerf-Less Removal of Si, Ge, and III–V Layers by Controlled Spalling to Enable Low-Cost PV Technologies. *IEEE J. Photovolt.* **2012**, *2* (2), 141–147.
